# Consent to research participation: understanding and motivation among German pupils

**DOI:** 10.1186/s12910-021-00661-z

**Published:** 2021-07-16

**Authors:** Jana Reetz, Gesine Richter, Christoph Borzikowsky, Christine Glinicke, Stephanie Darabaneanu, Alena Buyx

**Affiliations:** 1 Diakonissenkrankenhaus, Department of Paediatrics, Knuthstraße 1, 24939 Flensburg, Germany; 2 Institute of Experimental Medicine, Division of Biomedical Ethics, Kiel University, University Hospital Schleswig-Holstein, UKSH, Campus KielArnold-Heller-Str. 3, Haus U35, 24105 Kiel, Germany; 3 Institute of Medical Informatics and Statistics, Kiel University, University Hospital Schleswig-Holstein, Brunswiker Str. 10, 24105 Kiel, Germany; 4 Ethics Commission, University Hospital Schleswig-Holstein, Arnold-Heller-Straße 3; Haus U 27, 24105 Kiel, Germany; 5 Institute of Medical Psychology and Medical Sociology, Kiel University, University Hospital Schleswig-Holstein Kiel, Preußerstrasse 1-9, 24105 Kiel, Germany; 6 Institute of History and Ethics in Medicine, Technical University of Munich, Ismaninger Str. 22, 81675 München, Germany

**Keywords:** Paediatric consent, Medical research, Informed consent, Understanding, Data protection, Therapeutic-misconception

## Abstract

**Background:**

The EU’s 2006 Paediatric Regulation aims to support authorisation of medicine for children, thus effectively increasing paediatric research. It is ethically imperative to simultaneously establish procedures that protect children’s rights.

**Method:**

This study endeavours (a) to evaluate whether a template consent form designed by the Standing Working Group of the German-Research-Ethics-Committees (AKEK) adequately informs adolescents about research participation, and (b) to investigate associated phenomena like therapeutic misconception and motives for research participation. In March 2016 a questionnaire study was conducted among 279 pupils (mean age 13.1 years) of a secondary school in northern Germany.

**Results:**

A majority of participants showed a general good understanding of foundational research ethics concepts as understood from the AKEK consent form. Nevertheless, our data also suggests possible susceptibility to therapeutic misconception. Own health concerns and pro-social considerations were found to be significant motivational factors for participating in research, while anticipation of pain lessens likelihood of participation. Advice from trusted others is an important decisional influence, too. Furthermore, data security was found to be a relevant aspect of adolescents’ decision-making process.

**Conclusion:**

Bearing in mind adolescents’ generally good understanding, we infer the lack of knowledge about medical research in general to be one source of therapeutic misconception. To further improve the quality of consent we propose a multi-staged approach whereby general research education is completed before an individual becomes a patient or potential participant. To the best of our knowledge this is the first German questionnaire-study addressing issues of informed consent in a large under-age sample.

**Supplementary Information:**

The online version contains supplementary material available at 10.1186/s12910-021-00661-z.

**“What is Known”**

- From about 12 years of age, Children are able to give informed consent based on understanding, however, therapeutic-misconception is prevalent.
- Pro-social notions are strong motivational factors for adolescents to participate in research.

**“What is New”**

- Data security is—alongside well-known influences like the advice of parents and doctors and avoidance of pain—an important consideration for paediatric research participants.
- Temporally disconnecting research education and participation, thereby establishing knowledge before the informed consent-process, could help lessen therapeutic misconception and may lead to improvement of the quality of consent.

## Background

Medical research involving children has been limited in the past, rendering up to 90% of drug prescriptions on paediatric wards off-label therapy [1]. The European Union acknowledged the need to improve on this situation in 2006 by implementing the Paediatric Regulation [2] and establishing the Paediatric Committee to support and regulate the authorisation of medicine for children. Consequently, it is ethically imperative to establish procedures and tools for paediatric research that protect children’s wellbeing and right to have a say in their participation [3–5].

As one’s ability to give informed consent is, by definition, dependent on one’s ability to understand the subject matter at hand [6], children under a certain developmental age are unable to give fully informed consent, i.e. make a legally binding decision. Therefore, parents are required to give consent in their children’s stead considering not only their wellbeing, but also their presumed wishes [7]. As children advance in age, most appreciate increased involvement in the medical decision-making process [8, 9]. Their right to be involved is undisputed both ethically and legally [10], but the legal age at which an adolescent is deemed able to give consent differs considerably within the EU [11], ranging from 14 to 18 years [12]. There is, however, consensus as to the minimal requirements of paediatric decision-making: a minor’s assent (non-legal agreement) to research participation or treatment must be gained in addition to their parents’ consent, and their dissent (disagreement) must be taken into account when reaching a final decision [13].

### Study aims and research questions

To harmonise paediatric research consent-procedures across Germany, the Standing Working Group of the German Research Ethics Committees (AKEK) advocates using a national template consent-form which aims to provide information for adolescents aged 12 to 16 and can be adapted to fit the study designs of most clinical trials [14].

Our study’s primary aim was to evaluate the AKEK template consent-form, focusing on whether the consent-form enables adolescents of this age group to understand major elements of informed consent, such as their right to withdraw, their right to have a say and the risks associated with trial participation, rather than assessing whether adolescents are generally capable of understanding these fundamental principles of research ethics.

A further focus was to investigate why adolescents may choose (not) to participate in research by asking participants their opinions on different study designs [15], and also to explore whether notions of therapeutic misconception (whereby therapeutic and scientific goals of research are mistakenly conflated or confused) [16] may affect this age groups’ decision making process.

To the best of our knowledge this is the first German study of this kind addressing issues of informed consent in a large under-age sample. We hope our findings will contribute to re-evaluating and improving the consent-process in paediatric research.

## Methods

### Study design

Participants were adolescents and thus require parental consent to participate in a research study. Therefore, we drew a convenience sample utilizing the structural advantages of a school setting. Original questionnaire items were designed to match the aims of our empirical study. Following approval of the study by the local ethics committee (D414/16, 01.01.2016), years 6 to 9 of a secondary school (Gymnasium) in northern Germany were approached for participation (in March 2016). Parents were asked to give written consent, and to answer questions regarding their child’s medical background. Of 450 parents, 315 (78.00%) consented to their child’s participation, with 279 children (88.57%) then completing the questionnaire (36 eligible children were not in class on the day the survey took place).

The survey was conducted in all classes at the same time during one school-lesson. Pupils were provided with the adapted consent-form giving information about a low-risk, fictitious clinical trial concerning a non-existent allergy suppressant (see Additional file 1: Information sheet). They were made aware that their decision to participate was voluntary and without academic consequences. Eligible participants were asked for oral assent. Teachers were instructed to collect the consent-form before distributing the questionnaire. The collected data were matched and coded to guarantee anonymity.

### Questionnaires

1. Parents were asked to fill in a questionnaire concerning their child’s socio-demographic data and medical background (see Additional file 2: Questionnaire parents).
2. Questionnaire for pupils: An age-appropriate questionnaire was developed with input from a paediatrician and a clinical psychologist, pre-tested to determine its appropriate length and to evaluate its general comprehensibility (see Additional file 3: Questionnaire children and adolescents).

The questionnaire consisted of three parts: (a) comprehensibility of consent-form, (b) research risk appraisal and notions of therapeutic misconception and (c) assessment of motivational issues.

- The first part comprised nine elements related to the template consent-form: its perceived length and subjective general comprehensibility were to be evaluated via Likert-scaled items, linked with two open questions allowing for personal criticism or concerns. Five multiple choice questions were utilised to test participants’ understanding of major components of informed consent (right to have a say, right to withdraw, purpose of the study, allocation of placebo and personal gain derived from study participation) as understood from the consent form.
- The questionnaire’s second part was interrogative, with four statement items focusing on risk appraisal and therapeutic misconception [16]. For each statement, participants were asked to choose one of three to five possible answers (multiple choice format).
- The third part comprised 19 Likert-scaled items assessing the motivational power of the opinions of particpants’ parents, doctors, and friends, as well as potential influencing factors such as painfulness, material incentives, pro-social reasons and data security.

## Research methods

Descriptive statistics were calculated using *IBM SPSS Statistics* version 23.0 for Windows [17]. For continuous variables, descriptive statistics include mean (M) and standard deviation (SD) as well as minimum (MIN) and maximum (MAX) values. For categorical and ordinal variables, frequencies and percentages for each category are displayed within contingency tables. For associations between categorical variables, Spearman’s rank correlation was used. To assess the statistical significance between proportions, non-parametric tests were used as appropriate to item scale characteristics, including χ^2^ test, Fisher’s exact test and the Mann–Whitney U test. *p* values < 0.05 were regarded as statistically significant.

## Results

### Participants

A very high response rate was achieved in the study (88.57%). The average age of the 279 participants was M = 13.10 (SD = 1.24, MIN = 10.00, MAX = 16.00) years with no statistically significant difference between the sexes (U test, *p* = 0.391). The pupils were composed of 61.65% female and 38.35% male.

Most participants had little or no experience with medicine and research: 83.15% reported seeing a doctor fewer than five times a year (N = 232), 87.10% claimed to have no chronic medical issues (N = 243), and 86.02% had no prior experience with medical research (N = 240).

### Comprehensibility of consent-form

Most participants declared the length of the consent-form to be ‘appropriate’ (53.76%, N = 150), while 41.94% (N = 117) thought it was ‘too long’ and 1.43% (N = 4) felt it was ‘too short’.

When asked to rate the comprehensibility of the consent-form, a majority of 86.91% (N = 245) rated it as ‘easily comprehensible’ or ‘very easily comprehensible’, while 12.19% (N = 34) declared the text to be intelligible only ‘in parts’, ‘hardly’ or ‘not at all’.

When asked about specific topics explained in the consent-form, a majority (averaging 88.11% through all age groups) correctly answered four of the five questions regarding: the final decision maker (80.29%, N = 224), the overall trial purpose (95.34%, N = 266), the right to withdraw (89.96%, N = 251) and the mode of placebo/drug allocation (84.23%, N = 235). By contrast, when asked about someone’s personal gain from study participation (according to the consent form) the answers were more divided, with most (57.75%, N = 161) believing that receiving the study medication will help them get better and 32.95% (N = 92) choosing “I have no personal benefit from study participation”, which was intended to represent the correct option.

### Contemplating research risk appraisal and notions of therapeutic misconception

When asked to compare the risk associated with participation in a medical study with the risk associated with an average medical consultation (when both involve the same procedure, e.g. taking medication or blood samples), 60.65% (N = 168) of the pupils judged study participation to entail a higher risk.

To investigate whether trial participation is perceived as being aimed at a study participant’s individual benefit, we posed two statements that required true/false answers. The first statement claimed that study participants will always receive the best possible treatment, which was disagreed with by 67.03% (N = 187). The second statement proposed that clinical trials are undertaken to improve participants’ health, and received almost equal amounts of agreement (48.75%, N = 136) and disagreement (47.67%, N = 133). When checking for overlap between those who agreed with either, neither or both statements, 35.84% (N = 100) of participants were found to disagree with both statements, 17.92% (N = 50) confirmed both statements and 41.94% agreed with only one of the statements (30.11% (N = 84) agreement statement one/disagreement statement two, 11.83% (N = 33) vice versa).

### Assessment of motivational issues

Pupils were asked to indicate how hypothetical scenarios might influence their decision to participate in research being given the options of (a) more inclined, (b) less inclined or (c) of no consequence to their decision. Motivational factors may be categorised into factors of direct personal impact (Table 1: transparent), advice of others (Table 1: light grey) and pro-social factors (Table 1: dark grey)**.**

**Table 1:**
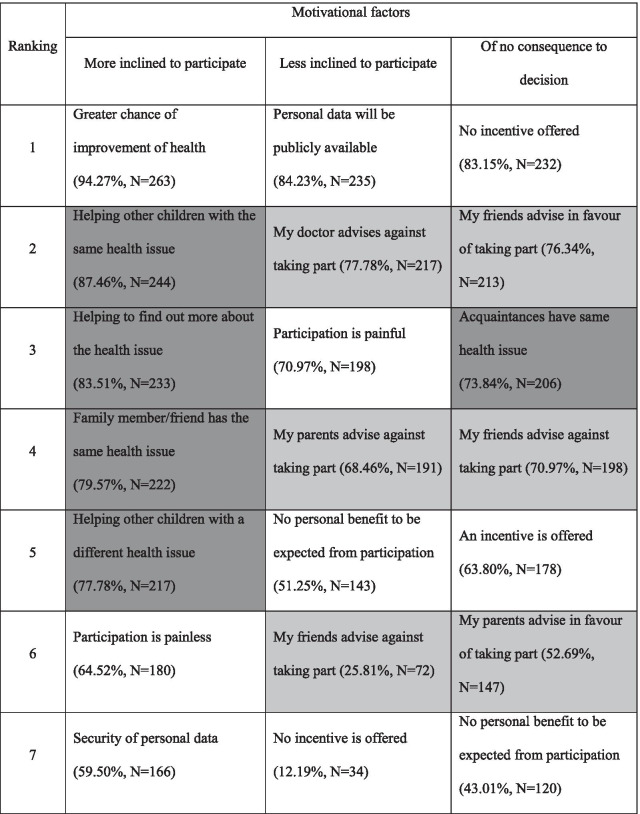
Ranking of motivational factors (transparent: direct personal impact, light-grey: advice of others, dark-grey: pro-social factors)

Analysis of motivational factors that resulted in greater inclination towards research participation revealed that ‘a greater chance of improved health’ as well as notions of solidarity and altruism had the strongest impact.

Regarding the motivational factors resulting in less inclination towards participation in a clinical trial, again, those scenarios that had a direct impact on the pupils themselves ranked highest, including public accessibility of data and the anticipation of pain. Personal opinions or advice from trusted others were also valued very highly.

The rankings of motivational factors are largely consistent through ages 10 to 16, excepting value placed on guidance by others and material incentives.

Younger age was linked to greater likelihood of being influenced by affirmative guidance, particularly that offered by parents (U test, *p* = 0.006) and doctors (U test, *p* = 0.039). Furthermore, parental guidance was found to be more highly valued by female pupils, displaying a statistically different response behaviour to that of males (χ^2^ test *p* = 0.001). (data not shown).

Secondly, the appeal of a material incentive appears to grow with increased age. More than 40.00% of 15-year-olds (40.54%, N = 15) would thus be motivated to participate in research compared to less than 10.00% of 11-year olds (6.67%, N = 2).

## Discussion

The need for more clinical research with minors is evident [1, 2], and was addressed in recent EU legislation. Although their right to have a say in the decision-making process is ethically and legally well established, consensus as to best practice in ensuring children’s and adolescents’ informed consent has yet to be reached (e.g., [18]). Interview studies (e.g., [19–23]), which are mostly qualitative and use smaller numbers of participants, have highlighted issues of concern.

Pioneering work by Ondrusek et al. [24] indicated that children younger than nine years old are likely to lack sufficient understanding to be able to assent. Tait et al. [25] found that understanding significantly improved after the age of 11. These findings were substantiated quantitatively using the MacArthur Competence Assessment Tool for Clinical Research (MacCAT-CR), demonstrating that adolescents from the age of 11.2 years are likely to be sufficiently competent to consent to research participation [26]. Our findings support this. Most of the 10- to 16-year-old participants in our study were capable of comprehending and correctly answering questions regarding their right to decide about their hypothetical study participation, their right to withdraw from study particpation, the overall purpose of the study to test a new medicine and the random allocation of placebo/treatment as understood from the consent-form.

A fifth item, that inquired about one’s personal benefit from study participation, gained a less uniform response. While we cannot be sure that the item was worded precisely enough to draw strong conclusions from, we do believe that the neat split in answers between those who thought that the hypothetical clinical trial was undertaken to improve participants’ health at least suggests a need to revise the parts of the consent form dealing with personal benefits. The contrasting responses show that study trials might be perceived as being primarily aimed at a study participant’s personal benefit by some participants. This in turn, could signal possibility of a certain degree of therapeutic misconception.

Notions of therapeutic misconception are difficult to overcome, even in conversation [27, 28]. Therefore, combining the established consent-process with supplementary tools (e.g. multimedia, [29–37]) is currently considered as a supportive measure. As the ability to successfully differentiate between research and treatment may, at its core, be related to conflicting cognitive frames, as suggested by Lidz et al. [30], they consequently advocate separating therapeutic from scientific research trials. Since this can be difficult to execute, increased general research education may also help alleviate therapeutic misconception.

Earlier qualitative work on potentially determining factors in children’s willingness to participate in research has implied that personal benefit [31], pro-social considerations [32] and advice from trusted others [33] are strong influences. Our study substantiates this with statistically significant findings for a large group of adolescents. This is in line with findings in adults [34, 35]. We also detected a distinct difference between older and younger adolescents, with the former more likely to agree to research participation if an incentive was offered, and the latter more inclined towards it if their parents or their doctors advised in favour. While both factors may indeed influence the decision-making process (for incentive see e.g. [36]), in our data incentives were not among the 7 strongest motivators for participation in research in any age group. Further research is needed to determine their relevance in different age groups.

We also found data security to be a relevant aspect of adolescents’ decision-making process. Facing a future of data-rich medical research, the future generation’s strong awareness of data security should be accommodated.

## Limitations

No validated tool was available to illuminate our research questions. Consequently, items were self-designed and therefore, their reliability and validity might be limited.

Instead of a random sample one of convenience was drawn to afford us the structural benefits of a school setting, e.g. being able to target the age group the AKEK consent form is intended for (namely, ages 12–16) by approaching years 6 to 9, who may on average be expected to fit this age bracket. Convenience sampling may potentially lead to biased results. However, this approach provided a rare opportunity to get a high response rate in a hard-to-reach research population. Further work is required to gain results with wider representability.

As our sample included largely healthcare and research-inexperienced adolescents, we also acknowledge that our findings might not be wholly transferable to all adolescents. Precisely though, because understanding of medical issues is linked to experience [37], we believe our findings to be relevant to future research practice. Not only has it been shown that adolescents with chronic illnesses and healthy adolescents share similar viewpoints on clinical trials [38], healthy adolescents may well be participants in non-therapeutic or foundational research, consequently consent-forms must cater for them, too. Sufficient understanding of the template consent-form by a relatively healthcare- and research-inexperienced minor is thus, in our eyes, a good indicator of its suitability for a wide range of study designs.

## Conclusion

Our study has shown that the template consent-form designed by the AKEK provides age-appropriate information allowing the envisioned age group to give informed consent. Our findings further strengthen the proposition that from the age of 12 years onwards adolescents are able to understand the main elements of a consent-form and to make an informed decision (e.g., [39]).

Pro-social motivation and mindfulness of potential discomforts (e.g., pain, embarrassment) are strong motivational factors that make adolescents more inclined to participate in research. However, when dealing with younger adolescents, researchers should be aware of their susceptibility to advice from trusted others when evaluating their consent, in order to ensure that their decisions are as unadulterated as possible [40].

As earlier studies have, we also found some indications of therapeutic misconception, which is a widespread phenomenon and has also been observed in research-experienced adolescents [21, 22] as well as in adult research participants (e.g., [35]).

Bearing in mind adolescents’ generally good understanding (e.g., [41]), we infer the lack of knowledge about medical research in general to be one source of therapeutic misconception. As understanding may improve further when the consent-process is temporally spaced out (e.g., [42]) we propose a multi-staged approach whereby general research education is ideally completed before an individual becomes a patient or potential participant. Placing general research education before patient specific consent-processes disconnects knowledge about characteristics of research from the actual project and may eventually improve the quality of consent.

## Supplementary Information

**Additional file 1: Information sheet.** Title of data: Information sheet for the information and consent of adolescents aged 11–16 years participating in a clinical trial of a medicinal product. Description of data: Information for the pupils about the hypothetical study.

**Additional file 2: Questionnaire parents.** Title of data: Dear parents, dear legal guardian. Description of data: Information for parents and legal guardians about the study including a declaration of consent and a short questionnaire concerning their child’s socio-demographic data and medical background.

**Additional file 3: Questionnaire children and adolescents.** Title of data: Questionnaire. Description of data: Questionnaire for the pupils consisting of three parts: (a) comprehensibility of consent-form, (b) research risk appraisal and notions of therapeutic misconception and (c) assessment of motivational issues.

## Data Availability

The datasets used and/or analysed during the current study are available from Jana Reetz on reasonable request.

## Abbreviations

AKEK: Standing Working Group of the German Research Ethics Committees
MacCAT-CR: MacArthur Competence Assessment Tool for Clinical Research
