# Supplementary material for: Consent to research participation: understanding and motivation among German pupils
Source: BMC Med Ethics. 2021 Jul 16;22:93. doi: 10.1186/s12910-021-00661-z (PMC8283995; doi:10.1186/s12910-021-00661-z)
Supplement: Supplementary file 1 — Additional file 1: Information sheet. Title of data: Information sheet for the information and consent of adolescents aged 11–16 years participating in a clinical trial of a medicinal product. Description of data: Information for the pupils about the hypothetical study. [file 12910_2021_661_MOESM1_ESM.pdf]

Information sheet

for the information and consent of adolescents aged 11-16 years participating in a clinical trial of a medicinal product

Dear student, in the following you will read an information sheet for a medical study. Please imagine that you have an allergy to pollen (hay fever), which causes you to have coughs, itchy eyes and skin rashes several months a year. Imagine that you are taking a medication ("allergy") that significantly improves these allergic reactions. Now imagine that you are asked to participate in the study described below, in which a new drug for your allergy is to be tested. We thank you!

Test center: Universitätsklinikum Schleswig-Holstein, Campus Kiel
Arnold-Heller-Straße 3,24105 Kiel
Testing medical doctor: .Herr Dr. Mustermann
EUDRACT-Nr. 2017-005071-80...........

Investigation of the effect of the allergy medication "Sternutio" in children and adolescents, which is already approved for adults
Testing plan code 1234-5678-9

Dear student,

we would like to ask you whether you are willing to participate in the clinical trial (study) described below.

Such studies serve research in medicine. They are necessary to find out more precisely whether and how well new drugs work or how well they are tolerated. The study that we present to you here is being conducted in several locations in Schleswig-Holstein; a total of about 500 children and adolescents are expected to take part.

You and your parents can decide whether you want to take part in the study. Your participation is voluntary, even if your parents agree; nobody forces you to participate. If you do not want to take part in the study or if you want to leave it later, you will not suffer any disadvantages.

You do not have to decide right away whether you want to participate; just take your time to think about it. This information should help you make your decision, because it is important that you understand everything. Your parents have also received written information from us. When you have read everything, you will talk about it. Your parents will certainly be able to answer most of the questions you have. And your doctor will also discuss everything with you about this study. He will have enough time to answer your questions.

1. Why is this study being conducted?

Up to now, your disease has been treated with the allergy medication "Allergi". By conducting the planned study we hope to achieve a better treatment with a more effective and better tolerated drug.

We want to test how the new drug, which is already approved and used for adults, also works in children. Drugs work differently in children and adolescents than in adults; the dosage cannot simply be halved. So we need to test how high the dosage of the drug has to be in children and young people and how it has to be used in order for it to work well. And we want to see how well children tolerate the drug, whether there are any side effects.

The investigational drug "Sternutio" is a drug in the trial phase, which means that it has not yet been approved by the authorities for the treatment of your disease.

2. Will I receive the new medicine in any case?

To enable us to better assess the effects and side effects of the new drug "Sternutio", all persons participating in the study will be divided into two groups. One group receives "Sternutio", the other group receives a so-called placebo for comparison.A placebo is a tablet that looks exactly like the drug but contains no active ingredient. Which group you belong to in case of your participation will be decided randomly, comparable to tossing a coin. The probability that you will receive "Sternutio" is 50%. In order to get a reliable result, neither you nor the doctor and your parents will know which group you belong to. However, if it is necessary, especially for your protection, your doctor will be able to determine which medicine you have received.

3. What is the course of the study and what do I have to consider when participating?

When you are accepted into this study, the history of your illness will be established and you will be given a comprehensive medical examination. This includes in particular a clinical examination (a doctor will examine you, for which you will have to take off all your clothes for a short time), the taking of blood and a measurement of your lung volume.

If you participate in the study, you must take one tablet unchewed with water in the morning and one in the evening before meals for the entire duration of the study, i.e. for four weeks.

It is important that you stop your current allergy medication two weeks before the start of the study.

For the duration of the trial, you must come to the clinic once a week.

At each visit we will take your blood, measure your blood pressure and perform a breath test to measure your lung volume. This will take about one hour at each visit. So you will have 4 visits with one hour each, which is 4 hours in total.

It is important that you keep to these visits:

It is important for you, as we use them to monitor your health while taking this new medicine, which has not yet been tested for its effectiveness in children and adolescents. If you feel unwell during the trial, it is important that you let us know. You can call us at any time.

And it is important for the success of our study. Only if we test the effect in all participating children and teenagers can we say how the drug works in children and teenagers.

After the end of the study "Sternutio" is no longer available for you. The study doctor will advise you on other treatment options.

Additional medication that your study doctor is not aware of may only be taken after consultation with him - except in emergencies. If you are treated by other doctors, you must inform them that you are participating in the study. You must also inform your trial doctor of any medical treatment you receive from another doctor during the trial. You will receive a study card that you should always carry with you in case of emergency.

4. What personal benefit do I get from participating in the study?

If you receive the new medicine, your symptoms may be relieved. However, as the efficacy of the new medicine has not yet been proven, it is also possible that you may not get the benefit you had hoped for. If you receive the placebo, you may not feel any effect. In this case your symptoms will not be relieved. If your symptoms lead to severe impairment of your health, you must immediately inform the study physician, who will then stop the study.

5. What are the risks associated with participation in the study?

Treatment with "Sternutio" can lead to adverse effects or discomfort. To date, the following common adverse effects and symptoms have been observed in adults for whom the drug is already approved:

- Fatigue (occasionally: more than one case in 100)

- Headache (occasionally: more than one case in 100)

- Nausea (rarely: more than one case in 1,000)

- Dizziness (rarely: more than one case of 1,000)

As with any new substance, the use of "Sternutio" can also cause new, previously unknown side effects.

Details of these adverse effects are described in the information sheets that your parents have received.

Interactions with other drugs are not known.

In addition, other measures implemented in the context of this study may involve risks or lead to complaints. Specifically, these are risks and burdens associated with blood collection. If carried out properly, this is associated with only minor risks. In rare cases, there may be increased bleeding from the injection site. In very rare cases, sustained damage to nerves or blood vessels is possible.

Please inform your doctor of any complaints, diseases or injuries that occur during the course of the study. If they affect you severely, please inform the doctor immediately, if necessary by telephone. Only then can we take the necessary countermeasures.

6. What other treatment options are available outside the study?

If you do not wish to participate in the study, your current allergy medication is also available to treat your disease.

7. Who may not participate in this study?

You may not participate if you are also participating in other studies or have recently participated. There should be at least eight weeks between them.

<u>Note for girls:</u>

If you are **pregnant,** you may not participate in this study.

At the beginning of the study, all girls must therefore undergo a pregnancy test. However, a pregnancy test can only reliably detect a pregnancy a few days after conception.

If you participate in this study, you must therefore use reliable contraceptive measures. Your doctor will tell you what these are.

The reason for this is that it has not yet been clarified whether "Sternutio" can cause damage to the unborn child if it is taken during pregnancy.

If you become pregnant during the trial or think you might be pregnant, you must inform your doctor immediately.

If you already have a baby and are breastfeeding, you **must not take part** in this study, as "Sternutio" could enter the baby's body with breast milk and cause damage to the baby**.**

Note to boys:

Boys who have sexual intercourse with girls may only participate in this study if effective contraception is assured. This is important, as the sperm cells which, together with the egg, make up the child can be damaged.

To prevent an unborn child from being damaged by the medication used in this study, pregnancy must be safely avoided. Your doctor will discuss with you what you can do to prevent pregnancy.

8. Am I insured during the study?

You are insured during the study against possible health damages caused by your participation in the study. Your parents have received more information about this. This also includes the question of what to do if you suspect that the study may have caused damage to your health.

9. Who decides whether I am to withdraw from the study?

You may terminate your participation in the study at any time, without giving any reason, without incurring any disadvantages in your medical treatment.

However, under certain circumstances it is possible that your participation may have to be terminated for other reasons, for example if you do not tolerate the new medicine well.

10. What happens to my data?

During the study, information about you and your illness will be written down and stored electronically. They are evaluated in a research institute. However, your name will not be given there, so that not everyone will learn something about you and your illness. However, certain persons, e.g. employees of the company that commissioned the study, may also learn your name and have access to the documents, especially if it seems necessary for your protection.

If you wish to terminate your participation in the study, the information already stored about you and your illness must remain stored.

Your parents have received further information on this.

11. What happens to my blood samples?

The blood samples will be used and stored after the study is completed in the following manner: They will be kept for three years and only made available to other researchers for further investigation.

If you want to end your participation in the study, you can decide whether you want your blood samples to be destroyed.

12. Who do I contact for further questions?

Consultations at the test centre
You will always have the opportunity for further consultations with the study doctor mentioned on page 1 or another study doctor.

Contact point
There is also a contact point at an authority that is available to you and your parents:

Bundesinstitut für Arzneimittel und Medizinprodukte
Fachgebiet Klinische Prüfung / Inspektionen
Kurt-Georg-Kiesinger-Allee 3
53175 Bonn
Phone: 0228 / 207-4318 Fax: 0228 / 207-4355
e-mail: klinpruefung@bfarm.de

Test Center: Universitätsklinikum Schleswig-Holstein, Campus Kiel
Arnold-Heller-Straße 3,24105 Kiel
Testing medical doctor: Herr Dr. Mustermann
EUDRACT-Nr. 2017-005071-80...........

Investigation of the effect of the allergy medication "Sternutio" in children and adolescents, which is already approved for adults
Testing plan code 1234-5678-9

Declaration of consent

The prerequisite for your participation is that you agree. If so, please sign this sheet. By doing so, you confirm to us that you wish to participate in the study and that you know that this is voluntary, that all your questions have been answered to your satisfaction and that you have had sufficient time to consider your participation. However, you can also say later at any time that you no longer wish to participate in the study. You will therefore have no disadvantages for your medical treatment.

I voluntarily agree to participate in the above mentioned study.

I agree to the collection and use of my data as described in the information leaflet given to me.

____

Name of the young person in block letters

__________________ ______________________

Place, date Signature of young person

I have conducted the educational interview and obtained the consent of the young person. I made sure that the young person understood everything, had no more questions and agreed to participate.

____

Name of testing medical doctor in block letters

____

Signature of testing medical doctor providing information
