# Supplementary material for: Consent to research participation: understanding and motivation among German pupils
Source: BMC Med Ethics. 2021 Jul 16;22:93. doi: 10.1186/s12910-021-00661-z (PMC8283995; doi:10.1186/s12910-021-00661-z)
Supplement: Supplementary file 2 — Additional file 2: Questionnaire parents. Title of data: Dear parents, dear legal guardian. Description of data: Information for parents and legal guardians about the study including a declaration of consent and a short questionnaire concerning their child’s socio-demographic data and medical background. [file 12910_2021_661_MOESM2_ESM.pdf]

Dear parents, dear legal guardian,

When children become ill, they should be given the best possible medical treatment. For many everyday childhood illnesses this can be guaranteed. However, especially for rare and serious diseases of childhood and adolescence, drugs are used for treatment, the effects of which have in some cases only been proven for adults. But adolescents are not "little adults". The treatment options based on calculated dosages and experience are therefore not adequate. Paediatricians need drugs that have been tested and proven to work on a child's body in order to be able to offer the best possible treatment to sick children and adolescents. Only through clinical studies, in which controlled tests are carried out with children, is it possible to increase the number of drugs approved for children. Minors are granted a decisive right when they give their consent to participate in a study. It is therefore essential that they understand the information about a study well. In Germany, there is a sample text for informing minors about participation in clinical trials.

In a research project of the Medical Ethics Group of the Christian-Albrechts-University of Kiel in cooperation with the paediatrician Dr. med. XXX, Germany, it is to be examined how well children understand this sample text and what should be improved if necessary.

With the approval of the Ministry for School and Vocational Training of the State of Schleswig-Holstein and the Ethics Comittee of the Medical Faculty of the University of Kiel, and with the kind support of the school management of the Helene-Lange-Gymnasium, we would like to have the patient information read by pupils and check their understanding by means of a short questionnaire.

The project will be integrated into the teaching of biology and will be limited to the scope of one school lesson. Prof. XXX, holder of the chair of medical ethics at the Christian-Albrechts-University of Kiel, will give a lecture for the children on the topic "Research in medicine".

Participation in our project is voluntary and has no effect on your child's ratings or grades. The questionnaires and information about the participating children are protected from access by unauthorized third parties. The evaluation is anonymous and the research results are published if necessary. The results will not be reported back.

To get an impression of the project, you will find the sample text and the questionnaire enclosed. We will distribute these documents to the participating children on the day of the survey. However, the children will not receive the sample text in the form in which you have it now. We are reworking it for a fictitious study on an allergy medication. This makes it easier for the pupils to put themselves in the situation. In order to obtain meaningful study results, we would like to ask you to

NOT discuss the contents with your child before the interview.

After the children have been interviewed, we will anonymise the questionnaires and destroy them after four weeks. It is possible to cancel participation within this period without giving reasons. Please send your revocation in written form to the project officer Mrs. XXX. She is also available to answer any further questions you may have.

If you agree to your child's participation, please confirm this below with your signature and include your child's consent in the parents' folder by the end of this week.

In the following we would also like to ask you some orienting questions about your child's experience with diseases and research, as this may influence his or her answers.

Declaration of consent

I hereby agree that

- my child participates in the above mentioned research project
- my details given below will be evaluated and published together with the results of the questionnaire in strictly anonymous form (without name, date of birth, gender etc.)

Date, Name in block letters Signature

Details of the participating child

Name of the child: Date of birth: __ . __ . ____

Gender of the child:

o female o male

This data is deleted immediately after the questionnaire has been entered

Approximate number of visits to the doctor by your child per year

o 3-5 o 6-10 o more

Does your child have a chronic illness, e.g. asthma, rheumatism or intestinal diseases?

o Yes o No

Are there any serious illnesses in your family that are known to your child?

o Yes o No

Has your child ever come into contact with medical research?

- o Yes, by the profession of family members
- o Yes, through participation in surveys
- o Yes, by

What experiences has your child had with hospitals in his life so far?

My child was a patient in the hospital:

o Never before o Yes, < 5x o Yes, > 5x

My child has already been to a hospital for another reason (e.g. to visit a friend or relative):

o No o Yes, < 5x o Yes, > 5x

Many thanks for your support!
