# Supplementary material for: Consent to research participation: understanding and motivation among German pupils
Source: BMC Med Ethics. 2021 Jul 16;22:93. doi: 10.1186/s12910-021-00661-z (PMC8283995; doi:10.1186/s12910-021-00661-z)
Supplement: Supplementary file 3 — Additional file 3: Questionnaire children and adolescents. Title of data: Questionnaire. Description of data: Questionnaire for the pupils consisting of three parts: (a) comprehensibility of consent-form, (b) research risk appraisal and notions of therapeutic misconception and (c) assessment of motivational issues. [file 12910_2021_661_MOESM3_ESM.pdf]

Questionnaire

Dear student, dear student,

Thank you very much for participating in our research project. We want to find out how well children and young people understand the information sheet you have just read. You will help us to improve this questionnaire.
It is important to us that children and young people understand the text, because they have the right to decide what they want: to participate in research or not. Doctors and parents must abide by this! So imagine you have a disease and the doctor asks you if you want to participate in a study. The study should help to research new drugs for your disease. The doctor does not only need the consent of your parents, but also your assent. So that you know exactly what it is about, there is an information sheet. Now we need your help to find out whether we have explained the information in this information sheet in a comprehensible way.

Please read the information sheet and give it back to your teacher.

We have some questions for you. Please answer them spontaneously, without thinking about it too much. There are no right or wrong answers and no grades.

What is your name?..
How old are you? .... years

Please tell us first how understandable you find the information sheet:

well understandable	partly understandable	difficult to understand	not understandable
☐	☐	☐	☐

Is there anything that is particularly difficult to understand? If so, please write this down.:

……

Do you have any questions that the information sheet did not answer? If so, please tell us which ones:
……

How would you estimate the length of the text?

too short	too long	just right
☐	☐	☐

Now we would like to ask you a few questions about the content of the information sheet.
Please tick as you understood it from the information sheet. What does it say?

Imagine that your parents want you to participate, but you don't want to. What does the information sheet say: who is allowed to make the final decision according to the law? Please tick only one box.

☐ My parents decide.

☐ I decide.

☐ The doctor decides.

☐ Do not know.

What does the information sheet say: what is the general purpose of the study? Please make only one cross.

☐ My illness should be researched.

☐ A new medicine is to be tested.

☐ I should get well again.

☐ Do not know.

If you decide now that you want to participate in the study - what happens if you change your mind later? What does the information sheet say? Please tick only one box.

☐ I can stop at any time with no consequences for me.

☐ I can stop at any time, but then I will not be treated any further.

☐ I cannot stop.

What does the information sheet say: how is it decided whether you will receive the new drug or the placebo (i.e. a tablet without an active ingredient)? Please make only one cross.

☐ My parents decide.

☐ I decide.

☐ The doctor decides.

☐ It is decided at random.

What does the information sheet say: what benefits do you get from participating in the study? Please make only one cross.

☐ If I get the new drug, I will definitely get better.

.

☐ If I get the placebo, I will definitely get healthy

☐ If I get the new medicine, my condition improves.

☐ If I get the placebo, my condition improves.

☐ I have no benefit from participating in a study.

Now we would like to know your own opinion on some things.

We will ask you questions about imaginary situations and would appreciate it if you honestly say what you think when you answer.

Again, there are no right and wrong answers and no grades.

How do you assess the risk of participating in a medical study compared to a normal visit to a doctor where something similar is done (e.g. taking medication, taking blood samples)? Please make only one cross.

☐ The risk of participating in a study is greater than that of receiving "normal" medical treatment.

☐ The risk of participating in a study is less than that of receiving "normal" medical treatment.

☐ The risk of participating in a study is just as high as that of corresponding "normal" medical treatment.

Please rate this sentence: As a participant in a clinical trial you automatically receive the best possible medical treatment.

☐ that's true

☐ this is not true

Please rate this sentence: Clinical trials are conducted to improve the health of study participants.

☐ that's true

☐ this is not true

Your best friend asks you to help decide whether he/she should participate in a clinical trial of a new drug. What advice would you give him/her?

☐ He/she should participate in the clinical trial.

☐ He/she should not participate in the clinical trial.

Why?

………

Suppose you had to decide whether you wanted to participate in any clinical trial, which could be about diabetes or genetic diseases, for example.
Wie würde Deine Entscheidung aussehen? Würdest Du teilnehmen? Bitte nur ein Kreuz machen.

Yes	More like yes	More like no	No
☐	☐	☐	☐

What would influence your decision? Please tick how you would decide in the following situations. Please mark each line with a cross.

**If….**	**Then…** I would rather opt for study participation	I would rather not participate in the study.	it would have no bearing on my decision g
… I would get a **thank you** (like more mobile/PC time, money)	☐	☐	☐
… I would **not** get a thank you (such as more mobile/PC time, money)	☐	☐	☐
… my parents would **be in favor of** my participation	☐	☐	☐
… my parents were **against** my participation	☐	☐	☐
… my doctor would be **in favor of** my participation	☐	☐	☐
… my doctor would be **against** my participation	☐	☐	☐
… my friends would be in **favor of** my participation	☐	☐	☐
… my friends would be **against** my participation	☐	☐	☐
… I would have a **better** chance of recovery through my participation	☐	☐	☐
… I gain **no benefit** from my participation	☐	☐	☐
… through my participation **more is found out about my illness**	☐	☐	☐
… through my participation **other children with the same disease** could be helped	☐	☐	☐
… through my participation **other children with the other diseases** could be helped	☐	☐	☐
… participation would be **painless**	☐	☐	☐
… participation would be **painful**	☐	☐	☐
…my personal information and results are **kept secret** (data security)	☐	☐	☐
… my personal information and results are **publicly accessible** (e.g. on the Internet)	☐	☐	☐
… someone in your **family or circle of friends** has the disease being investigated in the study	☐	☐	☐
… **someone you know** (e.g. a celebrity) has the disease being investigated in the study	☐	☐	☐

Is there anything else that is important for you to decide for or against participating in a study? If so, please write down here what that is:

……

Now please imagine that you have decided to participate in any medical study. You can help to find out more about the human body, but you are not helping yourself or anyone you know.

Would you be willing to do or have the following things several times during the study?
Please make a cross in each line:

Cheek swab (with a cotton swab is stroked once from the inside over your cheek)

Yes	More like yes	More like no	No
☐	☐	☐	☐

Abdominal ultrasound (a picture of your body from the inside; this does not hurt, but you must lie still on your back for a few minutes while the doctor presses on your naked stomach with a kind of pen

Yes	More like yes	More like no	No
☐	☐	☐	☐

Clinical examination (a doctor will examine you, for which you will have to take off all your clothes)

Yes	More like yes	More like no	No
☐	☐	☐	☐

CT (a picture of your body from the inside; it doesn't hurt, but there is a radiation exposure for your body and you have to lie still in a rattling tube for some minutes)

Yes	More like yes	More like no	No
☐	☐	☐	☐

Taking blood (a needle is poked into your arm)

Yes	More like yes	More like no	No
☐	☐	☐	☐

Now please imagine that you let a researcher examine your blood for a study. But he doesn't need everything he took from you. What would you like him to do with the rest of your blood? Here you may make several crosses if you find several answers to be correct.

☐ He shall destroy the rest of my blood.

☐ He may use the rest of my blood for his other research projects.

☐ He may give the rest of my blood to other researchers.

☐ He shall store the rest of my blood, and shall ask me if it shall be used again.

☐ He may do whatever he wants with my blood

Please tell us why you checked that:

……

Many thanks for your participation in our research project.
